# Mice Deficient in GEM GTPase Show Abnormal Glucose Homeostasis Due to Defects in Beta-Cell Calcium Handling

**DOI:** 10.1371/journal.pone.0039462

**Published:** 2012-06-28

**Authors:** Jenny E. Gunton, Mary Sisavanh, Rebecca A. Stokes, Jon Satin, Leslie S. Satin, Min Zhang, Sue M. Liu, Weikang Cai, Kim Cheng, Gregory J. Cooney, D. Ross Laybutt, Trina So, Juan-Carlos Molero, Shane T. Grey, Douglas A. Andres, Michael S. Rolph, Charles R. Mackay

**Affiliations:** 1 Diabetes and Transcription Factors Group, Garvan Institute of Medical Research, Sydney, Australia; 2 St Vincent’s Clinical School, University of New South Wales, Sydney, Australia; 3 Faculty of Medicine, University of Sydney, Sydney, Australia; 4 Department of Diabetes and Endocrinology, Westmead Hospital, Sydney, Australia; 5 Division of Immunology, Garvan Institute of Medical Research, Sydney, Australia; 6 Department of Physiology, University of Kentucky College of Medicine, Lexington, Kentucky, United States of America; 7 Department of Pharmacology and Toxicology, Virginia Commonwealth University School of Medicine, Richmond, Virginia, United States of America; 8 Department of Molecular and Cellular Biochemistry, University of Kentucky College of Medicine, Lexington, Kentucky, United States of America; 9 Division of Diabetes, Garvan Institute of Medical Research, Sydney, Australia; 10 Institute for Glycomics, Griffith University, Brisbane, Australia; 11 Department of Immunology, School of Biomedical Sciences, Faculty of Medicine, Nursing and Health Services, Monash University, Clayton, Australia; 12 Department of Pharmacology and Brehm Diabetes Center, University of Michigan Medical School, Ann Arbor, Michigan, United States of America; Universitat de Barcelona, Spain

## Abstract

**Aims and Hypothesis:**

Glucose-stimulated insulin secretion from beta-cells is a tightly regulated process that requires calcium flux to trigger exocytosis of insulin-containing vesicles. Regulation of calcium handling in beta-cells remains incompletely understood. Gem, a member of the RGK (Rad/Gem/Kir) family regulates calcium channel handling in other cell types, and Gem over-expression inhibits insulin release in insulin-secreting Min6 cells. The aim of this study was to explore the role of Gem in insulin secretion. We hypothesised that Gem may regulate insulin secretion and thus affect glucose tolerance *in vivo*.

**Methods:**

Gem-deficient mice were generated and their metabolic phenotype characterised by in vivo testing of glucose tolerance, insulin tolerance and insulin secretion. Calcium flux was measured in isolated islets.

**Results:**

Gem-deficient mice were glucose intolerant and had impaired glucose stimulated insulin secretion. Furthermore, the islets of Gem-deficient mice exhibited decreased free calcium responses to glucose and the calcium oscillations seen upon glucose stimulation were smaller in amplitude and had a reduced frequency.

**Conclusions:**

These results suggest that Gem plays an important role in normal beta-cell function by regulation of calcium signalling.

## Introduction

Regulated insulin secretion from pancreatic beta-cells is required for normal glucose homeostasis. However, the molecular pathways underlying glucose-stimulated insulin secretion (GSIS) are complex and remain incompletely understood. In order to secrete insulin in response to glucose, beta-cells must sense a rise in extracellular glucose which in turn leads to a stimulus-secretion coupling cascade that triggers insulin granule exocytosis. Glucose is ‘sensed’ via an increase in the ATP to ADP ratio [1]–[2]. Glucose enters the beta-cell via facilitative glucose transporters [3]–[4], and is then metabolised via the glycolytic pathway, Krebs cycle and the electron transport chain (ETC) to generate 34–36 molecules of ATP per molecule of metabolised glucose [5]. Increased ATP:ADP closes K(ATP) channels, leading to membrane depolarisation [6]–[8]; this in turn leads to the opening of voltage-dependent calcium channels (VDCC) in the cell membranes, facilitating calcium influx into the beta-cell [9]–[11]. Insulin granule trafficking and insulin exocytosis in beta-cells are ATP and calcium dependent processes [12].

Beta-cells express a broad range of VDCC, but there is general agreement that L-type VDCCs are the major subtype that regulate insulin secretion [13]–[15], although other subtypes have been shown to contribute [16]. Thus, deletion of the Ca_v_1.2 isoform in murine beta-cells decreased calcium currents by ∼45%, and led to glucose intolerance in the whole animal [17]. While other VDCC such as Ca_v_2.3 are also involved, their role appears to be less prominent. In human islets, L-type calcium channel blockers impair beta-cell function [16], although other low and high voltage calcium channel isoforms are also expressed in human islets [18] and may also play important functional roles.

A number of interacting molecular systems, including phosphorylation of the pore-forming subunit of the channel by numerous kinases or interaction with calmodulin, can modulate VDCC activity and the resulting Ca^2+^ currents. Recent studies in cardiomyocytes and neurons have highlighted a role for members of the RGK (Rem/Rem2/Rad/Gem/Kir) subfamily of the Ras-related small GTPases in VDCC regulation [19]–[20]. The RGK subfamily differ from the majority of Ras superfamily GTP-binding proteins in lacking a C-terminal lipidation/prenylation membrane anchoring domain, and they are subject to transcriptional regulation [19]. Recent structural studies suggest that these proteins might not operate as guanine nucleotide-induced molecular switches [21].

Individual RGK family members differ in their tissue distributions. However, when overexpressed, all have the ability to inhibit Ca^2+^ current from voltage-dependent Ca^2+^ channels in a Ca_V_β-dependent manner [22]–[24]. In addition, Gem and Rad can associate with and modulate Rho kinase β activity (ROKβ), to regulate cytoskeletal dynamics [25]. Gem was the first member of the RGK family to be identified, and subsequent studies showed that it is expressed in a diverse range of cell types, including pancreatic beta-cells [22], [24], [26]. By over-expressing the gene *in vitro*, the molecular mechanisms by which RGK family members regulate VDCC are starting to emerge [19]. For instance, a previous report demonstrated that Gem over-expression in Min6 beta-cells inhibited glucose-stimulated insulin secretion, consistent with what is known about the importance of Ca^2+^ channels in triggering beta-cell insulin secretion [24].

Based on the expression of Gem in pancreatic beta-cells, the effects in Min6 cells, and the ability of Gem to regulate the function of VDCC in neurons, we hypothesised that decreasing Gem may improve insulin secretion and glucose tolerance *in vivo*. To explore the physiological role for Gem in beta-cells and to address the hypothesis that Gem-mediated regulation of Ca^2+^ channel regulates glucose-stimulated insulin release, Gem-null mice were created and characterised.

## Materials and Methods

### Ethics Statement

All animal work has been conducted according to relevant national and international guidelines. All studies were approved by the Garvan Animal Ethics Committee.

### Generation of Gem-deficient Mice

**Figure 1:**
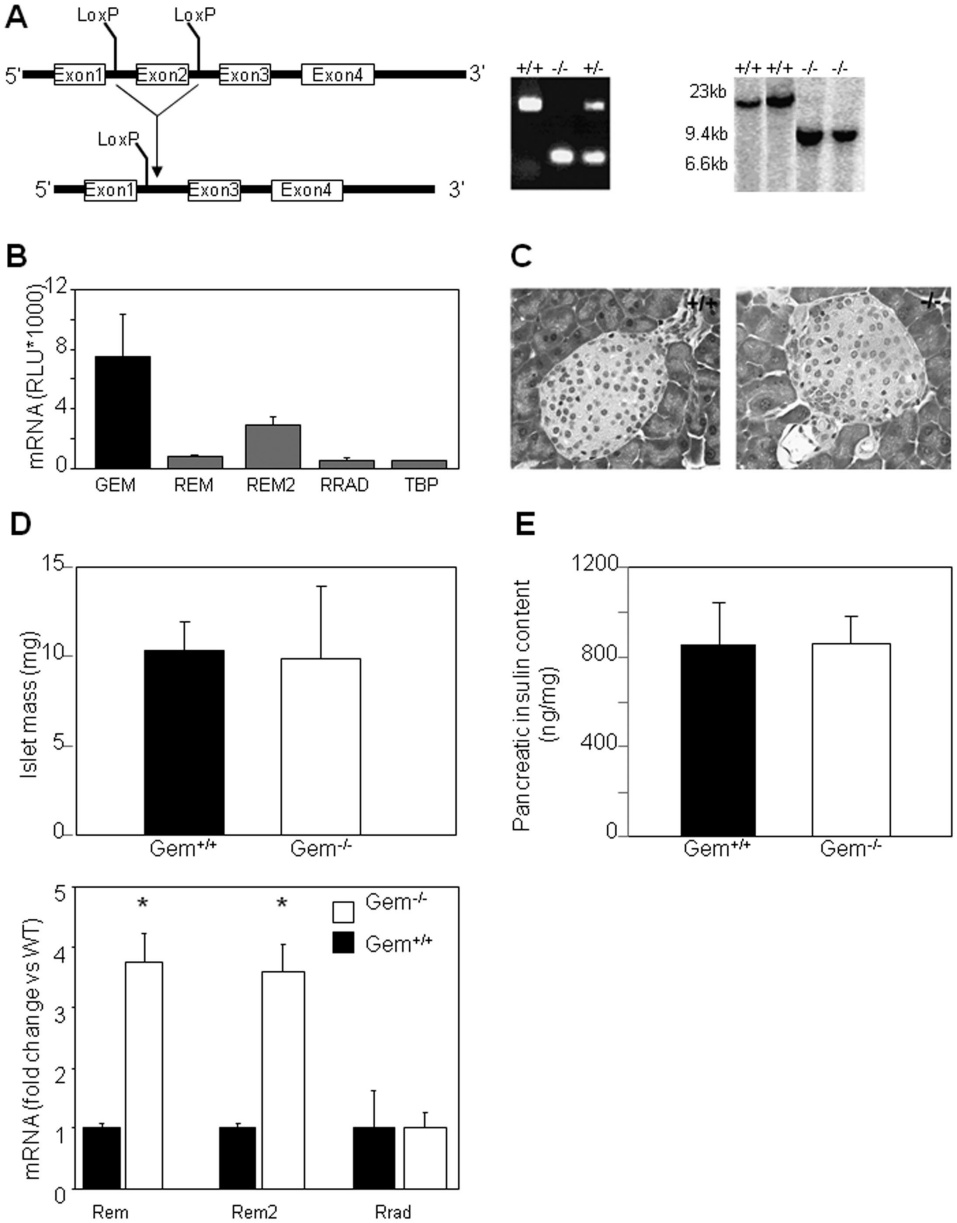
Gem deletion does not alter islet development . **A**) Lox-p sites were introduced on either side of exon 2 of the Gem gene. Deletion of exon 2 was confirmed by PCR and by Southern blotting. **B**) *GEM* mRNA is highly expressed in human islets compared to other members of the RGK family and to the house-keeping gene TATA-box binding protein (TBP). **C**) Islet morphology and islet mass (**D**) were not altered by Gem deletion in mice. **E**) Total insulin content of the pancreas was not altered by Gem deletion. Black bars indicate Gem^+/+^ mice, and white bars indicate Gem-deficient (−/−) mice. Error bars indicate ±1SEM.

Conditional Gem-deficient (Gem^−/−^) mice were generated on a C57BL/6 background by Ozgene (Perth, Australia). The targeted deletion of Gem was achieved by disruption of exon 2 of the Gem gene (Fig. 1A, B). The targeting construct, which contained two loxP sites flanking exon 2, was subsequently cloned into a PGKneo vector and transfected into mouse embryonic stems cells. Transfected embryonic stem cells were then microinjected into C57BL/6 blastocysts to generate chimeras that were bred with C57BL/6 mice. To delete exon 2, Gem^flox/flox^ mice were bred with “Deleter” mice carrying the Cre transgene [27]. The Cre transgene was bred out from the line to obtain both Gem^−/−^ and Gem^+/+^ (wild-type) mice.

Deletion of exon 2 was confirmed by sequencing Gem transcripts from Gem+/+ and Gem^−/−^ cDNA (primers ATC ACA CAG CCT CGG ACT GC and GAA TGA GAG GAG GCT GGC CTA) using conventional approaches at SUPAMAC (University of Sydney, Australia).

### Glucose Tolerance Tests (GTT)

GTT were performed in 8 week old male mice that were fasted overnight. Blood was collected from a tail nick at the times indicated. An intraperitoneal (i.p.) injection of glucose (2 g/kg) was given after the 0 minute time point. Glucose was measured as previously reported [28].

### Glucose Stimulated Insulin Secretion (GSIS)

GSIS was performed as previously reported [18] in mice aged ∼12 weeks. Mice were fasted overnight. Blood was collected from a tail nick at time 0, then mice received 3 g/kg of i.p. glucose, and further blood samples were collected for insulin ELISA.

### Insulin Tolerance Testing (ITT)

To assess whole body insulin sensitivity, insulin tolerance tests were performed as previously reported [29] in mice at 16 weeks of age. Mice were fasted overnight, and were given 0.5 U/kg of insulin i.p. then blood glucose was measured at the times shown.

### Isolation of Primary Murine Islets

Islets were isolated from 8–10 week old male mice as previously described [28], [30]. A Ficoll density gradient at 4°C was used to separate the islets from other tissue.

Isolated islets were hand-picked and 15 islets were placed into each microcentrifuge tube. Islets were washed twice and then pre-warmed DMEM containing low glucose (1 mM glucose) was added for 2 hours to return insulin secretion to baseline. Then baseline insulin secretion was measured in fresh low glucose media. The islets were then stimulated with the glucose concentrations indicated for 15 minutes. After washing in PBS, islets were lysed in acid ethanol for measurement of total insulin content. Insulin was measured by ELISA (Crystal Chem) or RIA (Lincoplex), according to the manufacturers’ protocols. ATP was measured using the Roche bioluminescence kit as we have previously reported [28].

### Pancreas Histology and Determination of Islet Mass

Formaldehyde-fixed whole pancreata from overnight-fasted mice were paraffin-embedded, cut into 5 µm sections and stained with haematoxylin and eosin (H&E). Islet mass was determined by calculating the islet area relative to total area, using 5 µm pancreas sections that were selected to be >100 µm apart. Sections (3–4 per pancreas) were analyzed to determine mean relative islet area per mouse, which was then multiplied by total pancreas weight to obtain islet mass. Proportional beta-cell area per islet was calculated using insulin staining as previously reported [18].

### Fura-2 Measurements

Islets were incubated with 2 µmol/l Fura-2 AM and 1 µl of 2.5% pluronic acid for 30 min at 37°C. After loading, islets were washed and incubated for 20 minutes in saline containing 115 mM NaCl, 3 mM CaCl2, 5 mM KCl, 1 mM MgCl2, 10 mM HEPES and 11.1 mM glucose (pH 7.2). [Ca^2+^]i was measured by placing islets in a small recording chamber on an Olympus IX50 inverted epifluorescence microscope (Olympus, Japan). Fura-2 was excited at 340/380 nm using a galvanometer driven mirror that alternated a light beam from a Xenon source (HyperSwitch; IonOptix, Milton, MA). A photomultiplier tube collected the 510 nm emission using IonWizard software (IonOptix). Solutions were perfused through the recording chamber using either a gravity fed system, or using a peristaltic pump. Free Ca^2+^ levels are expressed as fura2 ratios in most cases, or the ratios were converted to absolute concentrations using an in vitro calibration curve, as in [31].

## Results

### Generation of Gem^−/−^ Mice

Mice with targeted disruption of the *Gem* gene were generated using the Cre-loxP recombination system (Figure 1A). Deletion of exon 2 was confirmed by Southern blot of genomic DNA (Fig. 1A, middle section), and by RT-PCR using cDNA generated from bone marrow (Figure 1A, right section). Sequencing of the full length and truncated transcripts confirmed insertion of a premature stop codon in the Gem^−/−^ mice. The sequence encoded by the truncated Gem^−/−^ deletes three of five guanine nucleotide binding sites, the calmodulin binding site, the domains required for interaction with ROKβ, and the motif for membrane localization [26], [32]. Gem^−/−^ mice were viable, fertile and of normal size and weight.

However, when heterozygotes were bred, the genotype proportions for male offspring deviated from those expected (χ2-test, *P*<0.009). From 280 pups born to 17 breeding pairs, an elevated proportion of Gem^−/−^ males (37%) was observed, with a reduced rate of heterozygous males (40%). Wild types were 23% of male offspring (p = ns). Mice otherwise appeared normal.

### Gem is not Required for Normal Pancreatic Islet Development

GEM and the other members of the RGK family are expressed in human pancreatic islets, with GEM exhibiting the highest relative level of expression (Figure 1B). As a first step in analysing beta-cells from Gem^−/−^ mice, we examined tissue sections for defects in pancreatic structure. Islet structure in Gem^−/−^ mice was indistinguishable from controls (Figure 1C) and calculated islet mass did not differ (Figure 1D). These data indicate that Gem is not necessary for the gross development of islets in the pancreas.

Whole pancreata were dissected after an overnight fast, and the insulin content per pancreas was measured. Gem^+/+^ and Gem^−/−^ pancreata contained similar amounts of insulin (Figure 1E), suggesting that insulin production and storage were not affected by Gem deletion. Proportional beta-cell area per islet was also not different (data not shown). In the Gem-null mice, there was increased expression of other family members Rem and Rem2 (Figure 1F).

### Gem^−/−^ Mice were Glucose Intolerant

**Figure 2:**
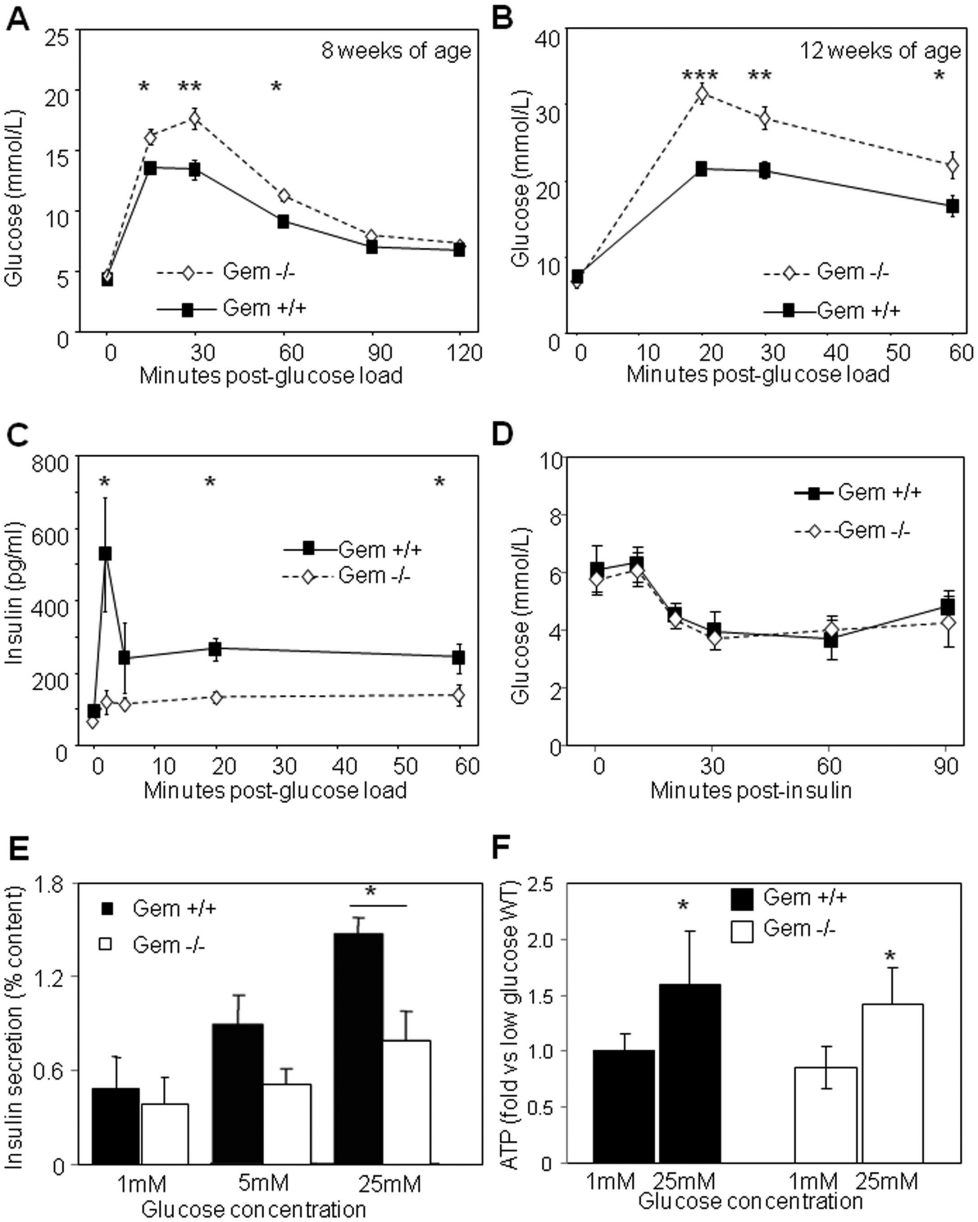
Gem-deficient mice were glucose intolerant. **A**) Eight (8) week old Gem^−/−^ mice had impaired glucose homeostasis on glucose tolerance testing (GTT). **B**) Glucose tolerance was also significantly worse in 12 week old Gem^−/−^ mice. **C**) Glucose stimulated insulin secretion in vivo was impaired in Gem^−/−^ mice. **D**) Whole body insulin sensitivity was un-altered in Gem^−/−^ mice, as indicated by insulin tolerance tests. **E**) Insulin release in isolated islets was impaired in Gem^−/−^ mice. **F**) Glucose stimulated increase in ATP content was normal in Gem^−/−^ mice. Error bars indicate ±1SEM. * = p<0.05, ** = p<0.01, *** = p<0.001.

Previous studies demonstrated that the forced over-expression of Gem in the Min6 beta-cell line inhibited glucose-stimulated insulin secretion [24]. Based on this, we hypothesised that systemic glucose homeostasis would be improved in Gem^−/−^ mice. Following an overnight fast, the fasting blood glucose levels of 8 week old null mice, were similar to wild-type controls (Figure 2A). However, Gem^−/−^ mice displayed significantly impaired glucose tolerance (p<0.005 by ANOVA for repeated measures, Figure 2A). The mice also had significantly increased area under the curve of GTT (p<0.002, data not shown). Glucose tolerance testing in a separate cohort of slightly older mice (12 weeks) was also significantly abnormal (Figure 2B).

### Impaired Glucose Tolerance in Gem^−/−^ Mice is Due to Decreased Insulin Secretion

Impaired glucose tolerance could result from impaired insulin production, impaired glucose-stimulated secretion (GSIS) or increased insulin resistance in liver and muscle. We hypothesised that the defect would be in GSIS since Gem is highly expressed in beta-cells but has not been reported to be expressed in peripheral insulin sensitive tissues. In addition, islet morphology was normal in Gem null mice.

Fasting insulin was not different in Gem^−/−^ mice, but post-challenge levels of insulin were significantly reduced compared to controls (Figure 2C). First phase and second phase insulin release were both significantly affected in vivo. Most striking, however, was the near complete ablation of first phase insulin secretion in the Gem^−/−^ mouse, which resembles the response of human patients with T2D who similarly lose first phase secretion [33].

### Insulin Tolerance Testing (ITT)

Although GEM has not been shown to be expressed in peripheral, insulin-sensitive tissues [34] it was important to determine whether the GEM nulls had altered whole body insulin sensitivity. We found no difference in the insulin sensitivity of wild type versus null animals whether insulin sensitivity was expressed in terms of absolute values of glucose (Figure 2D) or as a percentage of baseline glucose (data not shown). As shown in Figure 2C, fasting insulin levels did not differ.

### Gem^−/−^ Islets Exhibit Impaired Insulin Secretion in Response to a High Glucose Challenge

To confirm that Gem mice had an islet defect, islets were isolated for testing of GSIS *ex vivo*. The islets were stimulated *in vitro* with 1, 5 or 25 mM D-glucose, and insulin secretion was measured using a standard static incubation protocol. There was no difference in insulin release at low glucose (1 mM, Figure 2E). However, there was a trend towards lower insulin secretion at 5 mM glucose, and a ∼50% reduction in insulin secretion in response to high glucose concentrations (25 mM glucose, Figure 2E), confirming that the secretory defect persisted in isolated islets. The glucose stimulated increase in ATP concentrations was normal in Gem-null islets (Figure 2F), indicating that a more distal defect was responsible for impaired insulin secretion.

### Ca^2+^ Handling by Gem^−/−^ Beta-cells is Impaired

**Figure 3:**
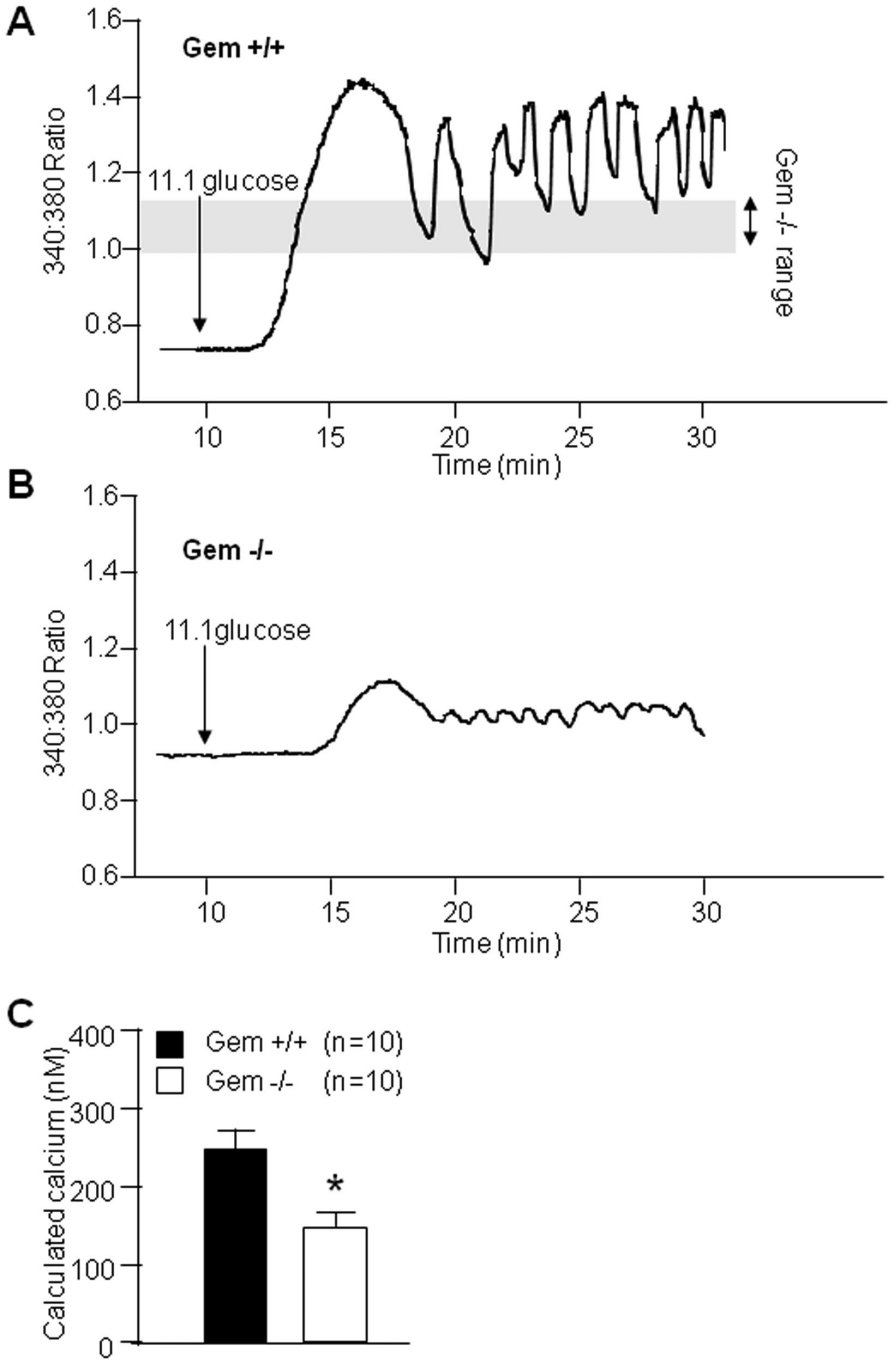
Gem-deficient mice have impaired calcium flux. **A**) Gem^+/+^ islets exposed to 11.1 mM glucose establish regular calcium oscillations. **B**) In contrast, islets from Gem^−/−^ mice have impaired oscillations. **C**) The calculated calcium at 11.1 mM glucose is significantly lower in Gem^−/−^ islets. Error bars indicate ±1SEM. * = p<0.05.

In pancreatic beta-cells, insulin release is Ca^2+^ dependent [35]. Since Gem is able to regulate Ca^2+^ channel function in other systems [24], we focused on Ca^2+^ as a potential mechanism underlying impaired GSIS in Gem^−/−^ mice. To test intracellular free Ca^2+^ handing, islets were loaded with the membrane permeable dye Fura-2-AM [36]. Islets incubated in 2.8 mM glucose after dye loading did not exhibit oscillatory activity, as expected. Following a glucose challenge, Gem^−/−^ islets failed to display the normal increase in Ca^2+^ concentration seen in wild type islets (Figure 3A), and the amplitude of the Ca^2+^ oscillations produced was decreased (Figure 3B). Calculated calcium concentration at 11.1 mM glucose was decreased by ∼50% in Gem-null islets. Thus, the glucose-stimulated Ca^2+^ response of Gem^−/−^ islets was depressed relative to controls, which could significantly contribute to the decreased insulin secretion seen in response to glucose both in vivo and in vitro.

**Figure 4:**
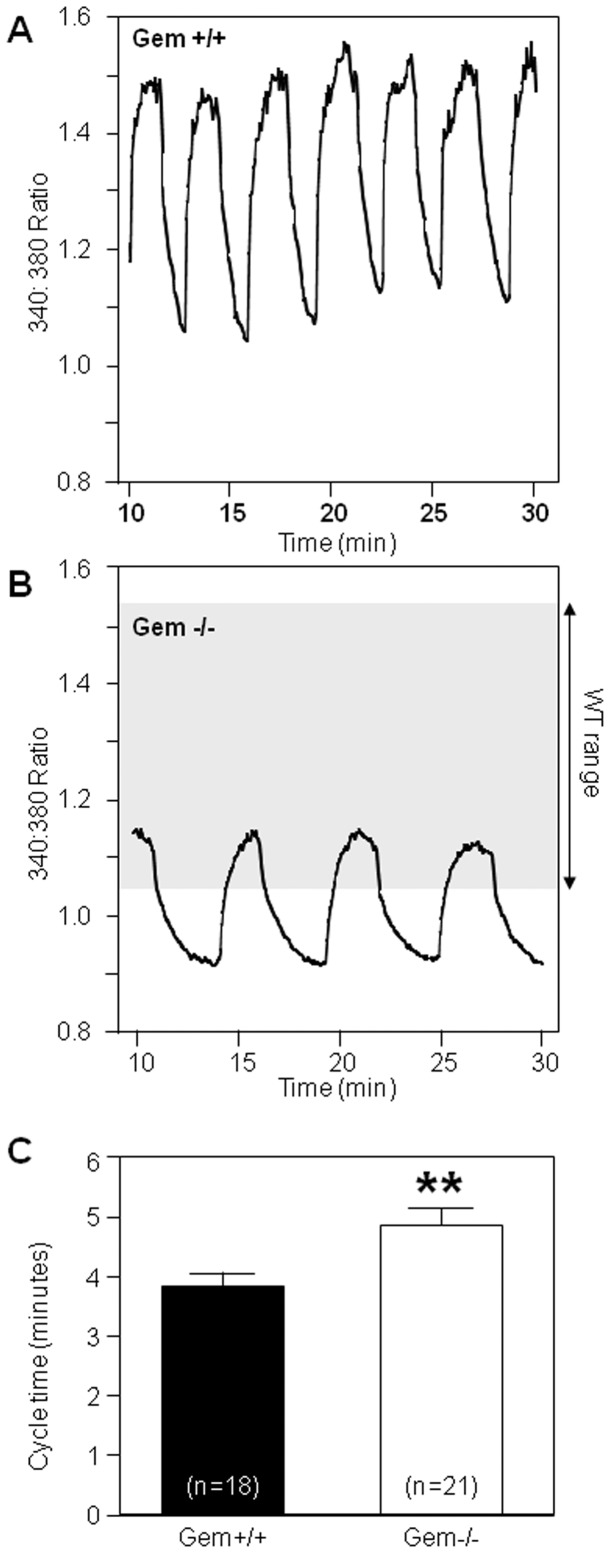
Calcium oscillations are slower in Gem^−/−^ mice. **A**) Wild-type mice (Gem^+/+^) have normal amplitude and frequency of calcium oscillations after exposure to 11.1 mM glucose. **B**) In contrast, Gem^−/−^ islets display smaller amplitude oscillations of lower frequency. **C**) Oscillation cycle time was slower in Gem-null islets. Error bars indicate ±1SEM. ** = p<0.01.

Under steady state conditions with 11.1 mM glucose, islets from Gem^+/+^ mice displayed regular [Ca^2+^]_i_ oscillations having a 3–5 minute period, as previously described for control islets [37] (Figure 4A). In contrast, the [Ca^2+^]_i_ oscillations of islets of Gem^−/−^ mice recorded under these same conditions had reduced amplitude (Figure 4B) and frequency, with their cycle time being ∼20% longer compared to Gem^+/+^ islets (Figure 4C). Thus, islets from the Gem^−/−^ mice had defective free Ca signaling compared to wild type controls, which may account for their abnormal insulin secretion.

## Discussion

The molecular pathways controlling GSIS are complex and calcium channel regulation in pancreatic beta-cells remains incompletely understood. In this study, we describe an important role for the Ras-related GTPase Gem in regulating glucose homeostasis, insulin secretion, and beta-cell calcium handling, including altered beta-cell [Ca^2+^]_i_ oscillations. Gem^−/−^ mice were glucose intolerant due to their impaired insulin secretion, which is likely to result, at least in part, from their markedly altered calcium handling properties. More detailed studies will be needed to more fully elucidate the contribution of particular ion channels and channel regulatory proteins in mediating this result.

Gem belongs to the RGK family of Ras-related GTPases, which includes Rad, Rem, and Rem2 [19]. While relatively little is known about the physiological roles of RGK family members, all RGK proteins are known to be capable of modulating VDCC function [23]–[24], with Gem and Rad also able to regulate cytoskeletal dynamics [32], [38]. These actions may be interrelated, as calcium has been shown to contribute to actin cytoskeletal dynamics [39]. The conserved ability of all RGK proteins to potently inhibit VDCCs, suggests that tissue-specific patterns of expression contribute to the functional differences between family members. Previous studies reported expression of Gem and Rem2 in rodent pancreatic beta-cells [22], [24]. Analysis of human islets indicate that multiple RGK family members are expressed in beta-cells, but further expression studies are required to focus specifically on beta-cells within the islet. Based on the dual expression of Gem and Rem2 in beta-cells, and their shared ability to modulate VDCC function, we speculate that there may be some redundancy between proteins in the regulation of GSIS. In this case, Rem2 and Rem were increased. They may partially compensate for the lack of Gem in Gem^−/−^ islets, even perhaps be deleterious, and it will be interesting to examine insulin secretion in islets from Rem2^−/−^ and Rem^−/−^ mice. Gem is expressed during differentiation and in the adult brain, and it is well known that the brain influences insulin secretion, especially in the anticipatory phase before a meal. Intra-peritoneal GTTs avoid this potential confounder, and the defect in insulin secretion persisted in isolated islets. However, a central contribution to glucose intolerance in these mice cannot be excluded.

The physiological function(s) of the RGK proteins remain incompletely understood despite a growing number of *in vitro* studies. The majority of this work has relied on RGK protein overexpression, with only a few studies attempting to address the physiological roles of endogenous RGK proteins. A growing number of studies have examined the function of Rad protein in the heart. Rad deletion generates mice with increased susceptibility to cardiac hypertrophy and more severe cardiac fibrosis following transverse aortic constriction induced pressure overload [40]. RNAi-dependent reduction of Rad in myocytes *increases* Ca^2+^ currents, providing evidence that Rad functions as a negative regulator of cardiac contractility, and that loss of Rad-dependent Ca^2+^ channel inhibition in the heart is pathogenic [41]. An RNAi screening approach identified a role for Rem2 in synaptogenesis [42], but the potential contribution of Rem2 to Ca^2+^ channel function was not assessed. Rem2 overexpression inhibits VDCC currents in primary hippocampal neurons [43]. It is not yet known whether all RGK proteins will demonstrate a dominant role in Ca^2+^ current regulation.

Importantly, none of these studies have examined the function of endogenous RGKs in pancreatic islets. Using a gene knockout approach, these studies identify Gem as an important regulator of glucose homeostasis. Gem is known to interact with the β-subunits of L-type Ca^2+^ channels, and to potently inhibit VDCCs [24]. Over-expression of Gem in MIN6 cells has been shown to decrease the surface expression of co-expressed VDCC Ca_V_α_1_ subunit, and to reduce human C-peptide release in cells expressing human pro-insulin [24]. Insulin secretion is linked to the extent of Ca^2+^ entry via L-type VDCC, so Gem-mediated blockade of L-type VDCC would be expected to, inhibit insulin secretion. Conversely, Gem knockout would be predicted to increase Ca^2+^ currents, and potentiate GSIS. In contrast, the present study shows that beta cells from Gem^−/−^ mice have decreased free Ca^2+^ oscillations, accompanied by decreased GSIS *in vivo* and *in vitro*. Nichols and colleagues presented an ‘inverse-U’ model to explain the secretory response to beta-cell excitability [44]. The crux of the ‘inverse-U’ model is that there is a linear direct-relationship between the insulin secretory response and excitability for low to mid-ranges of excitability. However, for relatively high levels of excitability, insulin secretion drops precipitously, a scenario that is exemplified by Kir6.2^−/−^ or SUR^−/−^ mice, which have tonic activation of L-type VDCC, yet have impaired insulin secretion [45]. We speculate that the Gem^−/−^ mice yield a similar, although not quite so extreme, pattern of GSIS as the K^+^-channel knockout mice because Gem^−/−^ directly increases tonic L-type VDCC. Alternative hypotheses, such as Gem deficiency affecting Ca^2+^ buffering by the cell, or the coupling between Ca^2+^ influx and granule release, remain possible.

In summary, the deletion of Gem in mice caused abnormal glucose tolerance that was due to a specific beta-cell dysfunction. Lack of Gem thus significantly diminished both the amplitude and the frequency of islet calcium oscillations seen following exposure to glucose. It is well known that GSIS results from K(ATP) channel mediated beta-cell depolarization and the subsequent opening of calcium channels and increased [Ca^2+^]_i_. Maintaining optimal levels of Gem activity thus appears to be important for preserving normal beta-cell function.
